# Advantageous Combinations of Nanoencapsulated Oregano Oil with Selected Antibiotics for Skin Treatment

**DOI:** 10.3390/pharmaceutics14122773

**Published:** 2022-12-12

**Authors:** Maya Margaritova Zaharieva, Mila Kaleva, Alexander Kroumov, Marta Slavkova, Niko Benbassat, Krassimira Yoncheva, Hristo Najdenski

**Affiliations:** 1 The Stephan Angeloff Institute of Microbiology, Bulgarian Academy of Sciences, 1113 Sofia, Bulgaria; 2 Faculty of Pharmacy, Medical University of Sofia, 1000 Sofia, Bulgaria

**Keywords:** *Staphylococcus aureus*, MRSA, oregano oil, nanoparticles, ciprofloxacin, gentamicin, hydrogels, skin application

## Abstract

The aim of the present study was to evaluate the antimicrobial activity of combinations between encapsulated oregano oil and the most commonly applied antibiotics (ciprofloxacin or gentamicin) against skin infections. In particular, chitosan-alginate nanoparticles loaded with oregano oil and the selected antibiotics were included in methylcellulose hydrogels. Consistency, spreadability, pH of the hydrogel and in vitro release rate of the oil were considered appropriate for topical application. The combination of encapsulated oil and gentamicin in the hydrogel resulted in a synergistic effect against methicillin-sensitive (MSSA) and methicillin-resistant (MRSA) *Staphylococcus aureus* strains. It was expressed in a fourfold reduction in the effective concentration of gentamicin and 98% inhibition of the bacterial metabolic activity. When ciprofloxacin was included in the combination instead of gentamicin, an additive effect with a two-fold decrease in the effective drug concentration and a 96% reduction in the bacterial metabolic activity were observed. Both combinations significantly inhibited the formation of MRSA biofilm by more than 90% when applied. In vivo application of the hydrogel containing the synergistic combination between the encapsulated oil and gentamicin did not induce irritation of the rabbit skin.

## 1. Introduction

Methicillin-resistant *Staphylococcus aureus* (MRSA) constitutes one of the major threats among antibiotic-resistant agents that cause deaths in the U.S with a health care cost of USD 3–4 billion annually [1]. MRSA can be isolated from mucus membranes, blood, urine, respiratory tract, sputum and other body fluids, but the prevalence is high in wounds [2]. As the *Staphylococcus aureus* remains the most common pathogen causing infection in wounds, the World Health Organization has stressed that this pathogen is one of the high priority multidrug-resistant organisms [3,4].

Therefore, the control of infection and prevention of MRSA is an attractive and important research topic [5]. Many studies have focused on the development of new antimicrobial drugs which are effective against multidrug-resistant (MDR) microorganisms through the combination of various active agents. The antimicrobial products originating from plants present a promising alternative; however, the reported antimicrobial activity is less than the classic antibiotics. So, herbal compounds alternatively can be used in combination with antibiotics aiming to enhance the activity against bacterial infections [6].

Aminoglycoside antibiotics are widely applied for the treatment of various types of bacterial infections because of their wide spectrum of activity. The spectrum includes Gram-positive (e.g., *Staphylococcus aureus*) as well as Gram-negative bacteria (e.g., *Pseudomonas aeruginosa*). That is why skin administration is an attractive approach to achieve a high antibiotic concentration at the infected site as well to avoid the negative effects of these antibiotics (e.g., nephrotoxicity and ototoxicity). However, an aminoglycoside resistance could occur, which has provoked the search for different combinations of these antibiotics with other antimicrobial agents. For example, Rodrigues et al. reported a synergy of gentamicin and the essential oil of *Croton zehntneri* suggesting that the oil acts as an adjuvant in the antibiotic therapy [7]. In another study, a synergistic effect of combination of citrus essential oils and gentamicin against methicillin-resistant *Staphylococcus aureus* was reported [8].

Fluoroquinolones (e.g., ciprofloxacin) are effective against a broad range of bacteria, including Gram-positive and Gram-negative bacteria. It is important to note that the wide spectrum of their activity could be beneficial for the direct treatment of skin infections and avoidance of their systemic effects. For example, the topical administration of ciprofloxacin could overcome the reported side effects of oral route such as tendon rupture, nerve damage or fluoroquinolone-associated disability [9]. Some studies have reported that ciprofloxacin exerted toxic effects on keratinocytes and fibroblasts in a time- and concentration-dependent manner [10,11]. However, the incorporation of ciprofloxacin in appropriate dermal drug delivery systems could be a useful tool to overcome eventual systemic and local undesired effects. For example, nanoemulgel (particle size of 203.2 nm) of linoleic acid and cellulose nanocrystals containing ciprofloxacin improved the antibacterial activity of the drug against the bacterial species *Staphylococcus aureus*, *Escherichia coli* and *Pseudomonas aeruginosa* and cytocompatibility on human dermal fibroblasts [12]. Ciprofloxacin-loaded PVP-foils and nanofibers had no effect on the overall skin metabolic activity and maintained drug concentration in the wound for 24 and 16 h, respectively [13].

The focus of the present study was to evaluate the antimicrobial activity of combinations of encapsulated oregano oil and antibiotics (ciprofloxacin or gentamicin) against skin infections caused by the bacterial pathogen *S. aureus*. Such combinations were considered advantageous aiming to decrease the minimal inhibitory concentration (MIC) of the selected antibiotics. The combinations were formulated in a hydrogel aiming to increase the residence time and to ensure the sustainable release of both compounds. The decrease in MIC reduced the risk of side effects, whereas the longer residence of hydrogel and nanosized particles with oregano oil additionally improved the efficacy of dermal treatment.

## 2. Materials and Methods

### 2.1. Materials

The oregano oil was obtained by distillation of *Origanum vulgare* L. which was cultivated and harvested in the southwest area of Bulgaria. According to the previously published phytochemical analysis performed by thin-layer chromatography and gas chromatography–mass spectrometry (GC–MS), the essential oil used in this study contained 39.44% o-cymene/m-cymene, 29.80% carvacrol, 20.82% terpinolene, 4.05% γ-terpinene, 3.23% thymol, 1.08% aromadendrene and 1.05% trans-β-ocimene/α-pinene [14]. Sodium alginate (very low viscosity) was supplied by Alfa Aesar GmBH & CoKG (Karlsruhe, Germany). The following reagents, chitosan (Mv 110,000–150,000), methylcellulose, absolute ethanol (#46139), PBS (Dulbecco’s Phosphate Buffered Saline, #D8537) and 3-(4, 5-dimethylthiazolyl-2)-2, 5-diphenyltetrazolium bromide (#M2128, MTT dye) were purchased from Sigma^®^ Life Science, Steinheim, Germany. The drugs gentamicin (#15750060) and ciprofloxacin (Ciproflav) were produced by Gibco (Waltham, MA, USA) and Warsaw Pharmaceutical Works Polfa S.A. (Warsaw, Poland), respectively. Both drugs were diluted in situ prior to each experiment in PBS and subsequently included into the hydrogel to reach the desired concentration. The working solution of gentamicin for the broth microdilution (BMD) and biofilm assays was 80 mg/L and that of ciprofloxacin was 10 mg/L. The media for cultivation of bacteria Trypticase Soy Agar/Broth (TSA/TSB, Himedia, India), Mueller Hinton broth (MHB, #M0405B), Mueller Hinton agar (MHA, #CM0337B) were purchased from Thermo Scientific-Oxoid (Hampshire, UK). Brain Heart Infusion broth and Brain Heart Infusion agar were delivered by Himedia (Maharashtra, India).

### 2.2. Preparation and Characterization of Methylcellulose Hydrogel Containing Oregano Oil-Loaded Nanoparticles

The encapsulation of the oregano oil into chitosan-alginate nanoparticles (OrO-NP) was performed following a previously reported procedure [14]. Shortly, a solution of oregano oil in methylene chloride was emulsified in a solution of sodium alginate, pre-gelled with calcium chloride and finally dropped in chitosan solution for complete electrostatic gelation.

Methylcellulose hydrogel (4% wt/wt) was prepared by gelation under heating and consequent cooling in a refrigerator. The methylcellulose was dispersed (700 rpm) in hot purified water (80 °C) and slowly cooled down at room temperature. Then, the remainder part of purified water was added in the form of exact amount of dispersion containing OrO-NP. The resulted viscous pre-gelled mixture was gently stirred for 30 min and stored in a refrigerator (4 °C) for complete homogenization.

Light microscopy of hydrogel samples was performed with a microscope Leica DM750 equipped with a camera ICC50W and Air Teach software (v.1.0.9874) (Heerbrugg, Switzerland). The estimation of the pH of empty and nanoparticle-containing hydrogels was performed by dipping an electrode of a pH-meter into diluted with distilled water samples. The consistency of empty and nanoparticle-loaded hydrogels was evaluated by the pharmacopoeial penetrometry test. The samples were transferred in the test container and the samples were stored at 25 ± 0.5 °C for 24 h. The gravity-driven standard penetrating object was released for 5 s and the penetration depth was measured and presented in millimeters as a mean of three determinations.

Spreadability of the hydrogel containing oregano oil-loaded nanoparticles (Oro-NP-HG) was determined applying the parallel-plate method [15,16]. Thus, 1 g of the hydrogel was placed between two glass plates (20 × 20 cm) and exact weights of 250, 500 and 750 g were put on the upper glass for 3 min. The diameter of the sample between the plates (d) was measured and its value was used for the calculation of the spreadability (1) and spreadability factor (2):

S = d^2^ × π/4, (1)

where S is the spreadability of the sample (mm^2^) and d is the diameter of the sample (mm).

Sf = S/P,(2)

where Sf is the spreadability factor of the sample (mm^2^/g) and P is the charging weight (g).

In vitro release of oregano oil was evaluated in a buffer medium (pH 5.5) using the dialysis method. Briefly, an appropriate amount of OrO-NP-HG was included in a dialysis membrane (MWCO 6–8 kDa, Spectra/Por, Spectrum Laboratories, Inc.) which was placed in a container that was filled with a 50 mL buffer. The release test was performed in a shaking water bath at 32 ± 0.5 °C. At predetermined time intervals (0.5, 1, 2, 3, 4, 5, 6, 7, 8 and 12 h) 2 mL of the released medium was withdrawn and replaced with a fresh medium. The amount of the released oregano oil was determined by UV-VIS spectrophotometry at a wavelength of 275 nm (Thermo Fisher Scientific, Waltham, MA, USA).

### 2.3. Bacterial Strains and Growth Conditions

The following *Staphylococcus aureus* strains were chosen for evaluating the antibacterial activity of OrO-NP included in hydrogel (OrO-NP-HG) and its combinations with two antibacterial drugs—gentamicin and ciprofloxacin: ATCC 29213 (penicillin and methicillin sensitive, MSSA) and NBIMCC 8327 (methicillin resistant, MRSA). The strains were maintained in Trypticase Soy Agar/Broth at 37 °C under aerobic conditions and cryopreserved using a Bacterial Culture Freezing System MAST CRYOBANK™ (Mast Group Ltd., Liverpool, UK).

### 2.4. Determination of Minimal Inhibitory and Bactericidal Concentrations

The minimal inhibitory concentration (MIC) of OrO-NP-HG was determined according to ISO 20776/1-2006 which refers to the broth microdilution method (BMD) [17]. First, a bacterial suspension with density 1.0 × 10^8^ CFU/mL (OD_600_) was prepared from an overnight liquid bacterial culture using a densitometer. It was further diluted to a working bacterial suspension (WBS) with a concentration of 5.0 × 10^5^ CFU/mL by adding of 50 µL of the first suspension to 10 mL MHB. Second, ten twofold serial dilutions of OrO-NP-HG were prepared in 96-well plates in volume of 50 μL/well starting from a concentration of 0.125%. Similarly, serial diluted solutions of gentamicin (20–0.039 mg/L) and ciprofloxacin (5–0.039 mg/L) were included in the hydrogels. Each concentration was replicated threefold. Gentamicin (20–0.039 mg/L) and ciprofloxacin (5–0.039 mg/L) included in hydrogels served as referent drugs for the positive controls. PBS was used as a negative control. Finally, 50 µL from the WBS was added to the OrO-NP-HG dilutions in the plates and they were incubated at 37 °C for 24 h. The lowest drug concentration, which inhibited visible bacterial growth, was determined as MIC. The requirements of EUCAST (European Committee on Antimicrobial Susceptibility Testing) were applied for MIC determination by evaluation of the obtained results [18].

After determination of the MICs, 100 µL of each sample from the BMD test representing the MIC concentrations and higher was sub-cultured in triplicate on MHA petri dishes with diameter 9 cm for 24 h at 37 °C. The lowest drug concentration that reduced colony growth of the treated bacteria by ≥99.9% was defined as the minimal bactericidal concentration (MBC).

### 2.5. Metabolic Activity Assay

After reading the results from the BMD test, the metabolic activity of the treated samples and the negative and positive controls was measured in the same plates using the MTT dye according to the protocol of Wang et al. [19] after minor modifications. This assay was applied for a quantitative evaluation of the antibacterial effects of OrO-NP-HG, ciprofloxacin and gentamicin. Briefly, each sample was mixed thoroughly to re-suspend the bacteria and MTT was added at final concentration of 0.5 mg/mL. The 96-well plates were incubated for 60 min at 37 °C. During the incubation the MTT dye was reduced by the membrane-located bacterial enzyme NADH: ubiquinone reductase (H^+^-translocation) to the colored product formazan (non-soluble violet crystals). The formazan crystals were then dissolved with an equivalent volume of 2-propranol containing 5% formic acid. The absorbance of the solution was measured at 550 nm/690_ref_ nm (Absorbance Microplate Reader L*x*800, Bio-Tek Instruments Inc., Winooski, VT, USA). The blank sample contained culture medium, MTT and the organic solvent.

### 2.6. Mathematical Modeling of Metabolic Activity after Single Drug Treatment

The inhibition effects of OrO-NP-HG and both antibacterial drugs were quantitatively evaluated using the results from the metabolic activity assay. The mathematical model of Lambert Pearson (LP) model (Equation (3)) was used to fit the experimental profile of the inhibition data [20] assuming that the influence of drugs on the microorganisms occurred on population level:

(3) $$Fa=\exp\left[-{\left(\frac{Dose}{P1}\right)}^{P2}\right],$$

where *Fa* represents the normalized maximum enzyme activity, (%); dose stands for the drug concentration; *P1* is the inhibitory constant which may be interpreted as IC_50_ in medical studies; and *P*2 stands for the slope of the curve.

The values of the model parameters *P1* and *P*2 were achieved with a non-linear identification procedure. The fitting of the experimental data was based on the weighted least square statistical method. The program was coded in the symbolic mathematics software MAPLE^®^ 15 (Maplesoft^®^, Waterloo, ON, Canada).

### 2.7. Biofilm Formation Assay

The protocol of Stepanovic et al. [21] was applied to evaluate the ability of the synergistic combinations to prevent biofilm formation of the MRSA strain. The combination effect was compared to that of the combination components after a single application. The test was performed in 96-well polystyrene tissue culture plates providing good conditions for the attachment of the bacteria to the surface of the wells. Briefly, twofold serial dilutions of the tested single hydrogel formulations and the combinations (also loaded in hydrogel) were prepared in BHI broth supplemented with 2% glucose (*w*/*v*) in a final volume of 100 µL/well. The bacterial inoculum was prepared the same way as described above for the BMD and an equivalent volume (100 µL) was added to each well. Cells were incubated at 37 °C for 24 h under static conditions. Thereafter, the supernatant was removed from each well and planktonic cells were removed by washing three times with PBS (250 µL/well). The remaining cells were fixed with methanol (200 µL for 15 min), air dried and stained with 0.1% crystal violet (200 µL/well). Excess stain was rinsed off with tap water and air dried. Biofilm formation was documented microscopically (40×). The dye bound to biofilm was re-solubilized with 160 μL of 33% acetic acid and the absorbance of each well was measured at 550 nm. The minimum biofilm inhibition concentration (MBIC_50_) was defined as the lowest concentration of the tested drugs that led to 50% inhibition on the biofilm formation. The biofilm inhibitory concentrations (BIC) were calculated in the program software GraphPad Prism (version 6.0.0 for Windows, GraphPad Software, San Diego, CA USA) with a mathematical model for the dose–response relationship (variable slope) after normalization of the data and logarithmic transformation of the applied concentrations (X-data).

### 2.8. Checkerboard BMD Test and Fractional Inhibitory Concentration Methodology

The checkerboard BMD test was used to determine the combination effects (synergistic, additive or antagonistic) between a range of four OrO-NP-HG concentrations (0.0039–0.03125%) and six concentrations of ciprofloxacin or gentamicin included in hydrogels (0.0156–0.5 mg/L). The two components of the combinations (OrO-NP-HG + CIP-HG and OrO-NP-HG + CN-HG) were serially diluted in a two-dimensional fashion to include 24 variants for each combination within a specified clinically relevant range for the antibacterial drugs. The test was performed and the result was reported according to the protocol for the BMD test described above.

The combination effects were calculated following the fractional inhibitory concentration (FIC) methodology. Synergy was defined as a four-fold decrease in the MIC of the drugs in combination (MIC_C_) when compared to the MIC after single application (Equations (4) and (5)). This ratio gives the FIC value for each combination component [22]:

(1) Step 1

(4) $$FIC\;\left(A\right)=\frac{MIC_{C}\;\left(A\right)}{MIC\;\left(A\right)}$$

(5) $$FIC\;\left(B\right)=\frac{MIC_{C}\;\left(B\right)}{MIC\;\left(B\right)},$$

where *FIC* stands for fractional inhibitory concentration, *A* stands for drug A (gentamicin or ciprofloxacin) and *B* stands for drug *B* (essential oregano oil).

The sum of both FIC values (Equation (6)) defines the type of the reported combination effect. Synergy is defined as Σ*FIC* ≤ 0.5, indifference—as 0.5 *<* Σ*FIC* ≤ 4, additive effect—as 0.5 *<* Σ*FIC* ≤ 1 and antagonism—as Σ*FIC* > 4 [21].

(2) Step 2

(6) ∑FIC=FIC (A)+FIC(B)

### 2.9. Skin Irritation Test

The potential of the synergistic combinations between OrO-NP-HG and ciprofloxacin or gentamicin to induce skin irritation was assessed on albino rabbits according to ISO 10993-10 [23]. The experiment was carried out with the permission of the National Ethics Committee for working with experimental rabbits (Nr. 232 for Animal House with Nr. 1113-0005, Ministry of Agriculture, Food and Forestry, Bulgarian Food Safety Agency, valid to 11th April 2024). Three healthy young albino rabbits with intact skin were acclimatized and cared for as specified in ISO 10993-2 [24] and Ordinance Nr. 20 (State Gazette of Bulgaria, Nr. 87, 09.11.2012). The fur on the back of the animals was clipped (10 × 15 cm) 4 h before the test. An amount of 0.5 g of each test or control material was applied directly to the skin and covered with an absorbent gauze patch. PBS and 10% SDS solution served as negative and positive controls, respectively. The application site was wrapped with semi-occlusive dressings for 4 h. The reaction of each application site was recorded at the 1st (±0.1) h, 24th (±2) h, 48th (±2) h and 72nd (±2) h after removing the non-occlusive bandage. The result was reported according to the scoring system for skin reaction in ISO 10993-10. The Primary Irritation Index (PII) was calculated based on the Primary Irritation Score (PIS).

### 2.10. Statistics

The statistical analysis (Student’s *t*-test) for the metabolic activity assay was performed using the software GraphPad Prism. A value of *p* < 0.05 was considered as evidence for a statistically significant difference. Each experiment was performed in triplicate. Minimum three replicates of each concentration, the positive, negative and untreated controls were tested within each experiment.

## 3. Results and Discussion

### 3.1. Incorporation of Oregano Oil-Loaded Nanoparticles in Hydrogel Form

**Figure 1:**
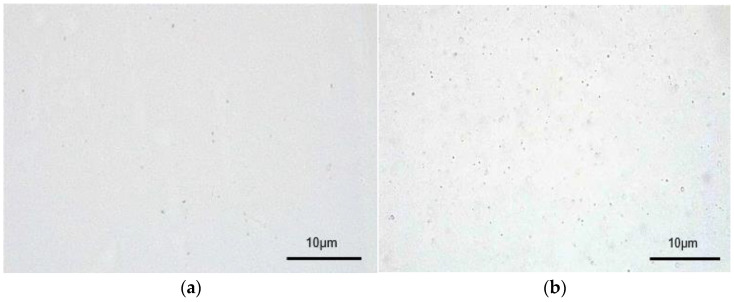
Light microscopy images (400×) of empty methylcellulose hydrogel (**a**) and hydrogel with oregano oil-loaded nanoparticles (Oro-NP-HG) (**b**).

The present study focused on the establishment of advantageous hydrogel formulations containing encapsulated oregano oil and selected antibiotics. The essential oils are powerful antibacterial compounds and a synergism with antibiotics could significantly improve the local therapy of skin infections without systemic side effects. The oregano oil was successfully entrapped in chitosan-alginate nanoparticles with a mean diameter of approximately 350 nm [14]. The small size of the nanoparticles was considered as a prerequisite for the more efficient transport of the loaded antibacterial compound and avoidance of efflux pump system of bacteria. Methylcellulose was selected as a gelling agent of hydrogel due to its non-ionic properties and high resistance against microorganisms. The distribution of nanoparticles in the hydrogel appeared to be homogeneous taking into consideration the light microscopic observations (Figure 1a,b). No phase separation or crystallization were observed.

**Figure 2:**
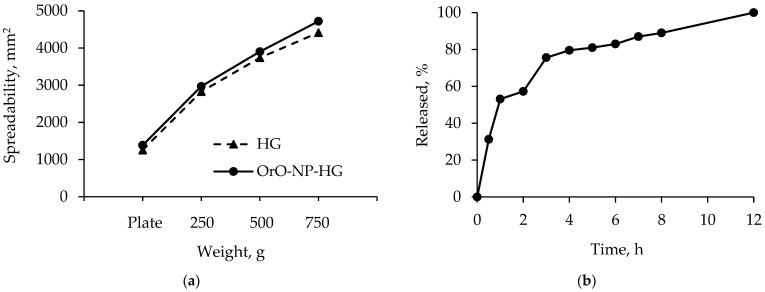
Spreadability of methylcellulose hydrogel (HG) and hydrogel containing the oregano oil-loaded nanoparticles (OrO-NP-HG) depending on the charging weight (**a**). In vitro release profiles of oregano oil from the hydrogel formulation (**b**).

For the preservation of nanoparticle structure, it was necessary that the pH value of the hydrogel be close to that of the nanoparticle dispersion. Our studies showed that the pH values of nanoparticle dispersion (4.72) and nanoparticle-loaded hydrogel were close, which was a prerequisite for preservation of nanoparticle structure (Table 1). Spreadability is an important feature of hydrogels ensuring uniform application and higher therapeutic efficacy. Some studies have reported changes in spreadability with the incorporation of nanoparticles. For example, the incorporation of clove and cinnamon oil in a chitosan/HPMC-based hydrogel increased the spreadability of the formulation [25]. In our study, the values of the factor of spreadability for empty and nanoparticle-loaded hydrogels revealed that the incorporation of oregano oil-loaded nanoparticles also increased spreadability (Table 1 and Figure 2a). The consistency was measured through the penetrometric method and the values are presented in Table 1. As seen, the empty hydrogel was characterized by a lower penetration value than the nanoparticle-containing hydrogel. Thus, the nanoparticle-loaded hydrogel possessed a higher penetration and slightly higher spreadability, indicating its appropriate consistency for skin administration.

An in vitro release test was performed in conditions that are typical for semi-solid dosage forms (pH = 5.5 of the buffer, 32 °C). The results showed a burst release of 50% in the first hours and a slower oil release after that (Figure 2b). The complete oil release was registered after 12 h. A similar profile with 100% released *Nigella sativa* oil for 8 h was observed from methylcellulose nanoemulgel [26]. Serra et al. [27] reported 45% released *Melissa officinalis* oil but the concentration of methylcellulose hydrogel was 10% (*w*/*v*). In the present study, the release profile of oregano oil was considered appropriate since the oil would be delivered to infected skin over 12 h.

### 3.2. Minimal Inhibitory Concentrations of OrO-NP, Gentamicin and Ciprofloxacin in Hydrogels

The minimal inhibitory concentrations (MIC) of OrO-NP, gentamicin and ciprofloxacin in hydrogels were determined as follows: (1) 0.0625 % OrO-NP and OrO-NP-HG for both strains; (2) 0.25 mg/L CIP and CIP-HG for the MSSA strain and 0.5 mg/L for the MRSA strain and (3) 0.25 mg/L CN and CN-HG for both strains. The values are given in Table 2. The MICs of CN were equal for both strains, whereas CIP was more effective on MSSA. All MICs achieved bacteriostatic effects as estimated after the overnight cultivation of aliquots from the samples on MHA. CIP-HG exerted bactericidal effect at concentration 2 mg/L, whereas for CN-HG the MBC was higher than 2 mg/L (higher concentrations were not tested in the experiments). The results showed that incorporation of the drugs and the essential oil into hydrogels did not change their efficacy as far as the estimated MICs remained the same.

### 3.3. Combination Effects of OrO-NP, Gentamicin and Ciprofloxacin in Hydrogels

The results from the Checkerboard assay from three combination ratios are presented in Table 3. The combination between OrO-NP-HG and CN-HG was labelled as Combo 1, and the other between OrO-NP-HG and CIP-HG as Combo 2. A synergistic effect was achieved by the combination of 0.0156% OrO-NP-HG and 0.0156 mg/L CN-HG, whereas the ratio 0.03125% OrO-NP-HG and 0.0625 mg/L CIP-HG led to an additive effect. If OrO-NP-HG is added to CN-HG in concentration of ¼ MIC, it diminishes sixteen-fold the MIC of the antibiotic which meets the criteria for strong synergism. Addition of ½ MIC OrO-NP-HG to CIP-HG decreased the MIC of the chemotherapeutic fourfold which is generally considered as additive effect. The combination effects on both strains were equivalent regarding the used concentrations despite the differences in the MICs of ciprofloxacin for MSSA and MRSA. It should be noted that the active concentrations of both antibacterial drugs were significantly diminished in the tested combinations with OrO-NP-HG—four- (MSSA) to eightfold (MRSA) for ciprofloxacin and sixteen-fold for gentamicin (both strains). This result proves the potential of OrO-NP to decrease the MICs of both drugs when used in combination with them thereby reducing the risk of adverse effects.

The concentrations used in the estimated synergistic and additive combinations are given in Table 4.

All combinations exerted a bacteriostatic effect as determined after overnight cultivation of aliquots from the samples on MHA. The MBC of the synergistic combination occurred at higher concentrations than the MIC_C_, namely—0.125 [%] OrO-NP-HG and 0.125 [mg/L] CN-HG. The combinations of 0.125 [%] OrO-NP-HG and 0.25 [mg/L] CN-HG were still bacteriostatic. Higher concentrations regarding both combinations were not tested in order to avoid higher percentage of the oregano oil which could cause skin irritations in higher doses [29].

### 3.4. Metabolic Activity of OrO-NP, Gentamicin and Ciprofloxacin and Combinations in Hydrogels

**Figure 3:**
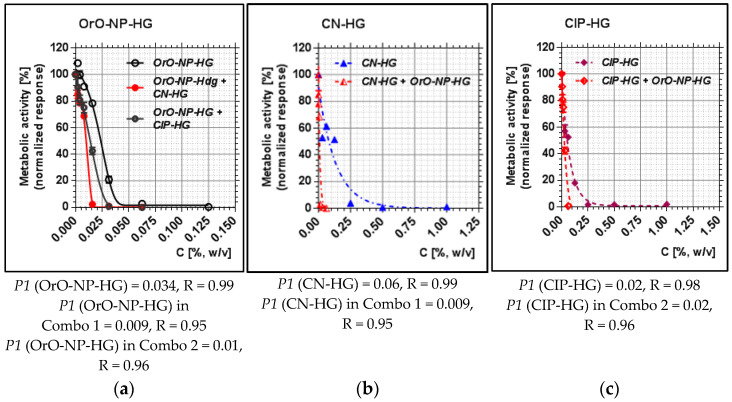
Metabolic activity of MSSA after treatment with OrO-NP, gentamicin and ciprofloxacin and synergistic combinations in hydrogels: (**a**) OrO-NP-HG, single application and in combinations with CN-HG or CIP-HG; (**b**) CN-HG, single application and in combination with OrO-NP-HG; (**c**) CIP-HG, single application and in combination with OrO-NP-HG. Legend: (**a**) OrO-NP-HG; (**b**) CN-HG; (**c**) CIP-HG; *P1*—inhibitory constant, which may be interpreted as the dose achieving 50% inhibition; R—correlation coefficient; OrO—oregano oil; NP—nanoparticles; HG—hydrogel; CN—gentamicin; CIP—ciprofloxacin.

The metabolic activity of OrO-NP, gentamicin and ciprofloxacin included in hydrogels was compared to that of the combinations regarding the median inhibitory concentrations (*P1*), representing inhibitory effect 50%. As presented in Figure 3, *P1* decreased by MSSA threefold (from 0.03 to 0.009) for OrO-NP-HG and six-fold for CN-HG (from 0.06 to 0.009) when both were applied in combination. As for the second combination, the median inhibitory concentration of OrO-NP-HG diminished threefold (from 0.03 to 0.01), whereas that of CIP-HG remained the same. The result reveals that Combo 1 was more efficient in decreasing the metabolic activity of the treated bacteria which is in correlation with the estimated synergistic effect. The median inhibitory concentration *P1* of gentamicin significantly decreased after the addition of OrO-NP-HG (Figure 3b), whereas *P1* for ciprofloxacin remained the same which confirmed the determined additive effect (Figure 3c). The *P1* values of OrO-NP-HG also decreased more in Combo 1 than in Combo 2 (Figure 3a) which corresponded to the results for both antibacterial drugs and combination effects.

**Figure 4:**
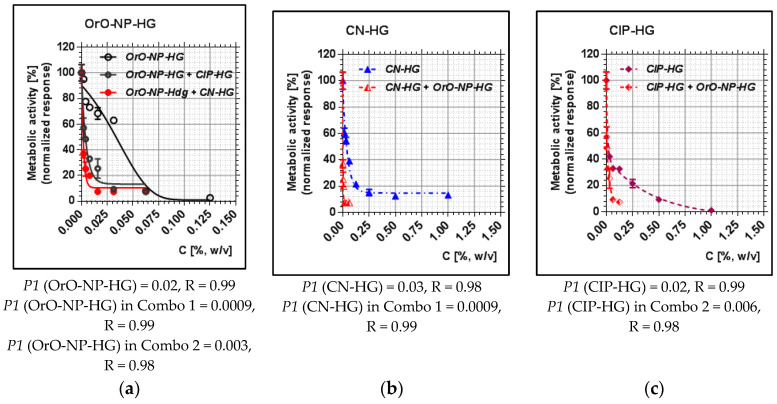
Metabolic activity of MRSA after treatment with OrO-NP, gentamicin and ciprofloxacin and synergistic combinations in hydrogels: (**a**) OrO-NP-HG, single application and in combinations with CN-HG or CIP-HG; (**b**) CN-HG, single application and in combination with OrO-NP-HG; (**c**) CIP-HG, single application and in combination with OrO-NP-HG. Legend: (**a**) OrO-NP-HG; (**b**) CN-HG; (**c**) CIP-HG; *P1*—inhibitory constant, which may be interpreted as the dose achieving 50% inhibition; R—correlation coefficient; OrO—oregano oil; NP—nanoparticles; HG—hydrogel; CN—gentamicin; CIP—ciprofloxacin.

Regarding the MRSA strain, the median inhibitory concentration of OrO-NP-HG dropped from 0.02 to 0.0009 (more than twenty-fold), that of CN-HG—more than thirty-fold (Figure 4). In the second combination, the value of *P1* for OrO-NP-HG decreased by six-fold, and that of CIP-HG—by threefold. Here, we observed the same trend in the effects of the single components and the combinations as by MSSA. However, the effect of Combo 2 on MRSA was more pronounced than on MSSA bearing in mind the stronger decrease in *P1* for CIP after the addition of OrO-NP-HG.

**Figure 5:**
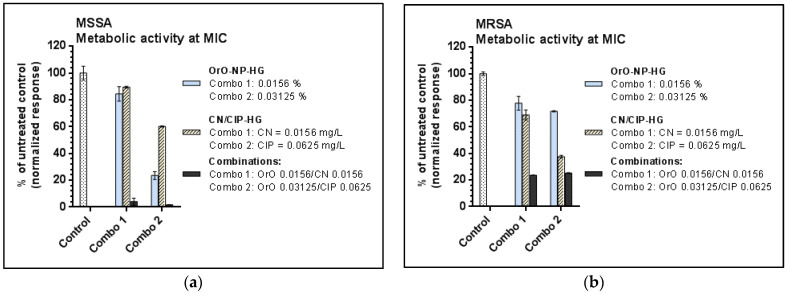
Metabolic activity of the test strains at the estimated minimal inhibitory concentrations—comparison between single treatment and combination effects: (**a**) metabolic activity of MSSA after exposure to OrO-NP-HG, CN-HG and CIP-HG or combinations; (**b**) metabolic activity of MRSA after exposure to OrO-NP-HG, CN-HG and CIP-HG or combinations. Legend: CN—gentamicin; CIP—ciprofloxacin; OrO-NP-HG—nanoparticles with oregano oil included in hydrogel.

The comparison between the metabolic activities of the treated strains at the MIC values is presented in Figure 5. As visible from the results both combinations inhibited significantly the vitality of the bacterial cells as follows: with 96% and 98% by MSSA for Combo 1 and Combo 2, respectively, and with approx. 75% by MRSA for both combinations.

### 3.5. Biofilm Inhibitory Concentrations of OrO-NP, Gentamicin and Ciprofloxacin and the Synergistic Combinations in Hydrogels

The concentrations used in the estimated synergistic and additive combinations for the biofilm evaluation are given in Table 5. The evaluation of the biofilm started for both combinations with MIC_C_ of OrO-NP-HG and the relevant antibacterial drug (CN or CIP) and was tested down to concentrations of 1/8 MIC_C_.

**Figure 6:**
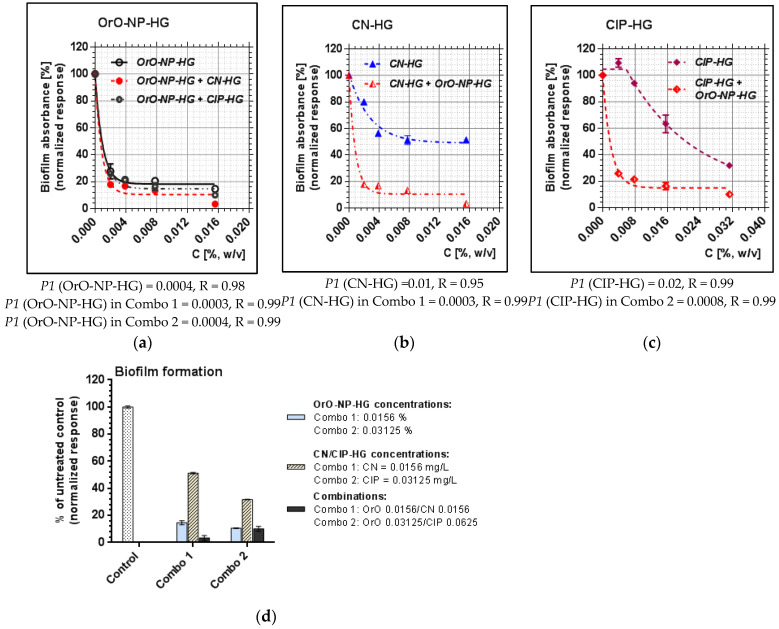
Inhibition of the biofilm formation by OrO-NP, gentamicin and ciprofloxacin or the synergistic combinations in hydrogels—absorbance of crystal violet: (**a**) OrO-NP-HG, single application and in combinations with CN-HG or CIP-HG; (**b**) CN-HG, single application and in combination with OrO-NP-HG; (**c**) CIP-HG, single application and in combination with OrO-NP-HG. The combinations in each point are given in Table 5. (**d**) The different values of *P1* denote the diminishment of the median inhibitory concentration of the hydrogel components as single application and in the tested combinations. Legend: *P1*, inhibitory constant, which may be interpreted as the dose achieving 50% inhibition; R—correlation coefficient; CN—gentamicin; CIP—ciprofloxacin; OrO-NP-HG—nanoparticles with oregano oil included in hydrogels.

The comparison between the biofilm formation between the treated groups of samples was evaluated quantitatively as percentage of the untreated control (Figure 6d). The 50% biofilm inhibitory concentration (BIC_50_) of OrO-NP-HG decreased only slightly in Combo 1 and remained the same in Combo 2. BIC_50_ of CN-HG decreased more than thirty-fold in Combo 1, and that of CIP-HG was diminished twenty-five times in Combo 2. The combinations inhibited the biofilm formation of MRSA as follows: with 98% (Combo 1) and 90% (Combo 2). The additive combination between OrO and CIP showed similar level of biofilm eradication from the surfaces of the treated wells as compared to the single effect of OrO applied in the same concentration, whereas the synergistic combination achieved almost full eradication. Full eradication of the biofilm was observed after application of combinations with concentrations 2MIC_C_ of the combination components.

**Figure 7:**
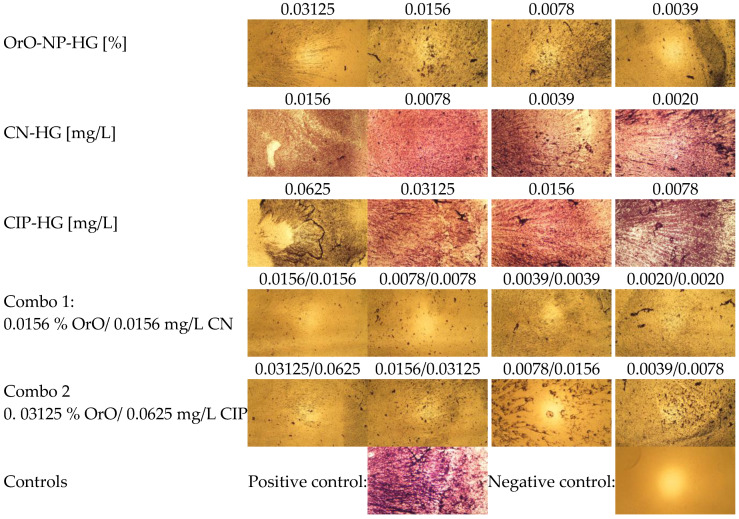
Biofilm formation after treatment of MRSA with OrO-NP, gentamicin and ciprofloxacin and the synergistic combinations in hydrogels (magnification 40×).

In Figure 7 the microscopic images (magnification 40×) of the biofilm formation after treatment with different concentrations of OrO-NP-HG, CN-HG, CIP-HG and the determined synergistic and additive combinations in serial dilutions are presented. The diminishment of the biofilm formation after incubation of MRSA with the selected combinations was visibly lower than after single treatment with the same concentrations.

### 3.6. Evaliuation of the Skin Irritaion Potential of Synergistic Combinations between OrO-NP and Gentamicin or Ciprofloxacin in Hydrogels

**Figure 8:**
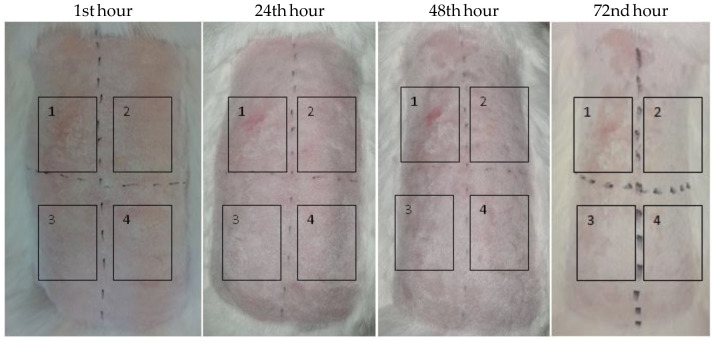
Skin irritation test—pictures of the experimental animals at the 1st, 24th, 48th and 72nd hour from the application of the patches. Legend: (1) 20 % SDS in PBS; (2) empty hydrogel; (3) OrO-NP-HG (0.0625%)/CN-HG (0.0625 mg/L); (4) osmotic solution of 0.9 % NaCl. The time interval is given in hours.

Empty hydrogel and the synergistic combination between OrO-NP-HG and CN-HG in ratio (0.0625% to 0.0625 mg/L), respectively, were applied for 4 h on rabbits and their dermal safety was assessed at the 1st, 24th, 48th and 72nd h after the exposition period (Figure 8). The positive control was 20% SDS dissolved in sterile distilled water, whereas the negative control was PBS. The Primary Irritation Score (PIS) and Primary Irritation Index (PII) for both test samples were equal to zero. In comparison, the PIS and PII of the positive control were equal to 3. The Cumulative Irritation Index (CII) was not calculated because a single exposure to the test samples did not induce skin irritation.

## 4. Conclusions

In conclusion, OrO-NP potentiated significantly the anti-staphylococcal activity of gentamicin and ciprofloxacin when applied simultaneously in a certain ratio in the form of hydrogel. The combination with the first antibiotic exerted a synergistic effect whereas the combination with the second one led to an additive effect. Both advantageous combinations inhibited the formation of the MRSA biofilm by more than 90%. The test for skin irritation confirmed the skin safety of the synergistic combination between OrO-NP and gentamicin. Altogether, the results obtained make the combinations an attractive option for further pharmacological studies aiming at the development of pharmaceutical formulations for the treatment of skin infections caused by methicillin-resistant staphylococci.

## Figures and Tables

**Table 1:** Comparison of the properties of empty methylcellulose hydrogel (HG) and hydrogel with oregano oil-loaded nanoparticles (Oro-NP-HG).

Sample	pH	Spreadability Factor, mm^2^/g	Penetration, mm
HG	5.21	8.22 ± 2.35	26.4
OrO-NP-HG	5.10	8.65 ± 2.24	36.0

**Table 2:** Minimal inhibitory concentrations of hydrogels with OrO-NP, gentamicin or ciprofloxacin after single application.

Bacterial Species	Minimal Inhibitory Concentrations				
	[mg/L]				[%]
	CN *	CN-HG	CIP **	CIP-HG	OrO-NP-HG ***
*S. aureus*	0.25	0.25	0.25	0.25	0.0625 ^§^
MRSA	0.25	0.25	0.5	0.5	0.0625

Legend: * Gentamicin, ** Ciprofloxacin, *** Oregano oil loaded nanoparticles included in hydrogel, ^§^ Data confirming previous investigations with nanoparticles [14,28].

**Table 3:** Combination effects between OrO-NP-HG and CN-HG or CIP-HG.

Strain	MIC_C-AB/CT_ [mg/L]		MIC_C-OrO_ [%]	FIC _AB/CT_	FIC_OrO_	∑FIC	Effect
*S. aureus*	CN-HG	0.0156	0.0156	0.0624	0.25	0.312	Synergism
	CIP-HG	0.0625	0.03125	0.25	0.5	0.75	Additive
MRSA	CN-HG	0.0156	0.0156	0.0624	0.25	0.312	Synergism
	CIP-HG	0.0625	0.03125	0.125	0.5	0.625	Additive

**Table 4:** Schema of the combinations between different concentration of OrO-NP-HG, CN-HG and CIP-HG tested on MSSA and MRSA.

Concentrations by Single Application:							
OrO-NP-HG [%, *w*/*v*]	0.1250	0.0625	0.03125	0.0156	0.0078	0.0039	0.0020
CN- or CIP-HG [mg/mL]	1.000	0.500	0.250	0.125	0.063	0.031	-
Concentrations in Combo 1:							
OrO-NP-HG [%, *w*/*v*]	0.0625	0.03125	0.0156	0.0078	0.0039	0.0020	-
CN-HG [mg/L]	0.0625	0.03125	0.0156	0.0078	0.0039	0.0020	-
Concentrations in Combo 2:							
OrO-NP-HG [%, *w*/*v*]	0.0625	0.03125	0.0156	0.0078	0.0039	0.0020	-
CIP-HG [mg/L]	0.125	0.0625	0.03125	0.0156	0.0078	0.0039	-

**Table 5:** Schema of the tested combinations on MRSA.

Concentrations by Single Application:					
OrO-NP-HG [%, *w*/*v*]	0.03125	0.0156	0.0078	0.0039	0.0020
CN-HG [mg/mL]	0.0156	0.0078	0.0039	0.0020	-
CIP-HG [mg/mL]	0.0625	0.03125	0.0156	0.0078	-
Concentrations in Combo 1:					
OrO-NP-HG [%, *w*/*v*]	0.0156	0.0078	0.0039	0.0020	-
CN-HG [mg/mL]	0.0156	0.0078	0.0039	0.0020	-
Concentrations in Combo 2:					
OrO-NP-HG [%, *w*/*v*]	0.03125	0.0156	0.0078	0.0039	-
CIP-HG [mg/mL]	0.0625	0.03125	0.0156	0.0078	-

## Data Availability

Data are available from the authors (see given emails).
